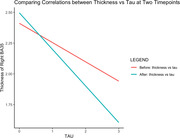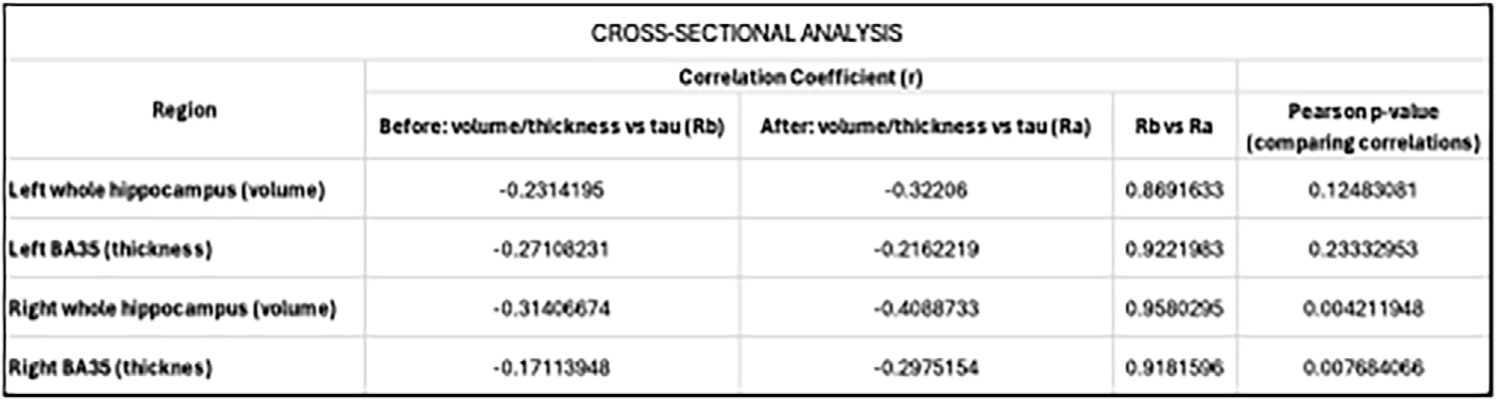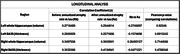# Does Neurodegeneration Lag Behind Tau Deposition in Alzheimer Dementia?

**DOI:** 10.1002/alz.094952

**Published:** 2025-01-09

**Authors:** Ujashi Shah, Long Xie, Emily McGrew, David A Wolk, Sandhitsu R. Das

**Affiliations:** ^1^ Perelman School of Medicine, University of Pennsylvania, Philadelphia, PA USA; ^2^ Siemens Healthineers, Princeton, NJ USA

## Abstract

**Background:**

Alzheimer’s disease (AD) is defined by the presence of ß‐amyloid (Aß) plaques and tau‐based neurofibrillary tangles (NFTs). Understanding the temporal relationship of NFTs with atrophy in early AD regions, specifically the medial temporal lobe (MTL), is critical for monitoring disease progression. Accumulation of NFTs has been suggested to precede atrophy because cross‐sectional measures of atrophy are more weakly associated with baseline tracer uptake than prospective longitudinal ones. However, longitudinal measures may be more sensitive to disease‐related atrophy at early AD stages. To address this, we examine cross‐sectional versus longitudinal measures before or after the time of tau PET.

**Methods:**

Data from patients with mild cognitive impairment (MCI) or dementia due to AD (n = 69, amyloid PET positive (Aß+) symptomatic adults) were analyzed. Tau PET date was the anchor point and MRI atrophy measures from earlier, ≤2 years before, and later, ≤2 years after, scans were derived. Hippocampal volumes and thickness of Brodmann area (BA) 35, the region of earliest tau accumulation, as well as their annualized rates of volume loss were extracted as atrophy measures. MTL tau was measured as tracer uptake in a combined BA35/ERC region. Cross‐sectional and longitudinal analyses included Pearson correlation between MTL tau and atrophy and Pearson and Filon’s z test to compare these correlations.

**Results:**

As expected, there was a significant correlation between atrophy and tau in the cross‐sectional analysis, which was generally stronger for later time points. Specifically, there was a statistically significant difference (p <0.05) for right hippocampal volume and BA35 thickness. In longitudinal data, while the correlation between annualized atrophy rate and tau was generally greater at both time points compared to the cross‐sectional data, these longitudinal correlations did not differ between the two time points.

**Conclusions:**

Here we show longitudinal atrophy rates before or after tau scans are similarly correlated. Later cross‐sectional measures have stronger correlations than earlier, likely due to more time to accrue atrophy relative to other sources of variation. These results question prior arguments of a lag between deposition and atrophy, which may be based on differences of sensitivity of cross‐sectional and longitudinal measures.